# Supplementary material for: Giant barocaloric effect in the ferroic organic-inorganic hybrid [TPrA][Mn(dca)3] perovskite under easily accessible pressures
Source: Nat Commun. 2017 Jun 1;8:15715. doi: 10.1038/ncomms15715 (PMC5461497; doi:10.1038/ncomms15715)
Supplement: Supplementary Information — Supplementary Figures [file ncomms15715-s1.pdf]

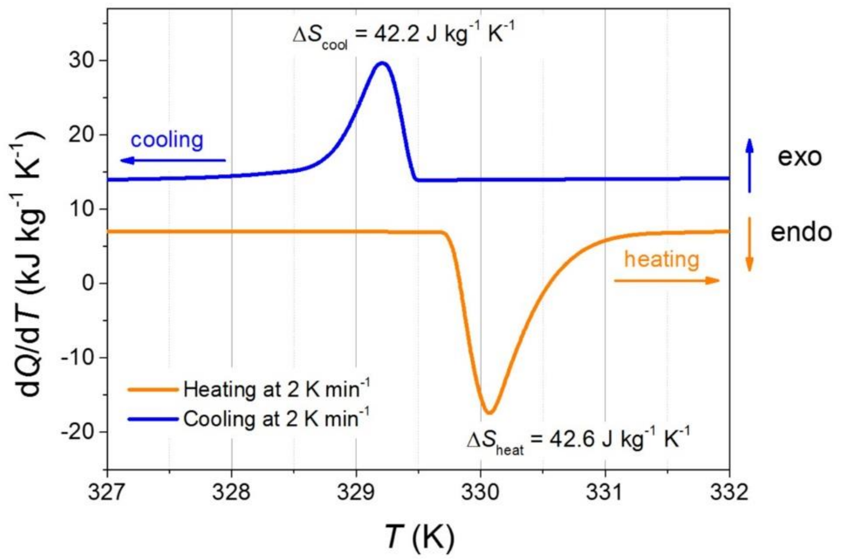

Supplementary Figure 1. Differential scanning calorimetry at ambient pressure. Heat Flow, $\delta Q/\delta T$ (kJ kg^{-1} K^{-1}), versus temperature curves, T (K), measured by DSC upon heating (bottom orange line) and cooling (top blue line) the sample [TPrA][Mn(dca)$_3$] at a rate of 2 K min^{-1}. ΔS_{heat} and ΔS_{cool} represent the entropy change related to the phase transition measured upon heating and cooling, respectively.

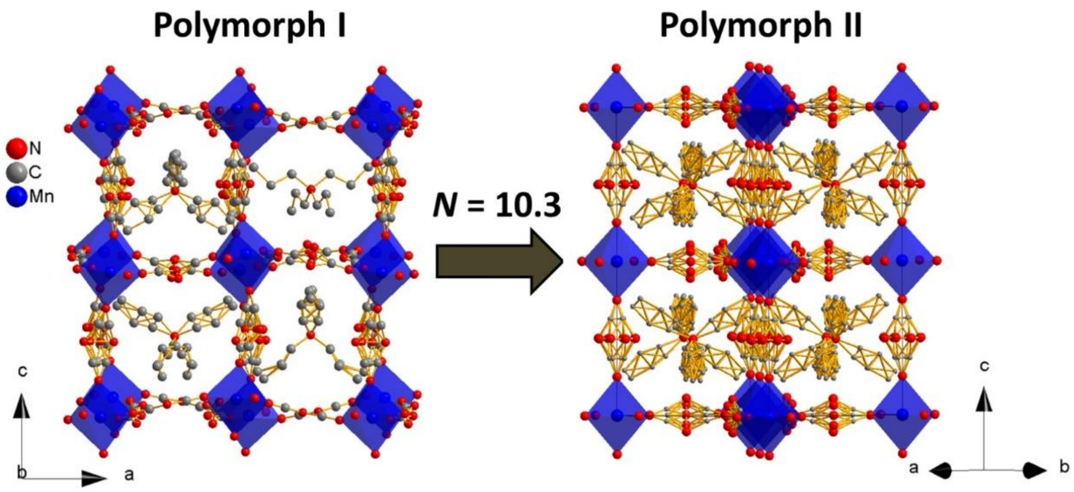

Supplementary Figure 2. [TPrA][Mn(dca)$_3$] polymorphs. Structures of the polymorphs I and II of [TPrA][Mn(dca)$_3$] obtained by single-crystal XRD and used for calculating the configurational entropy associated to the phase transition using the expression: $\Delta S_{conf.} = R \ln(N)$, with $N = (n_2/n_1)$, where n_2 and n_1 are the number of configurations in the two polymorphs. Note: the H atoms of the TPrA cations have been removed to facilitate visualization of the structure.

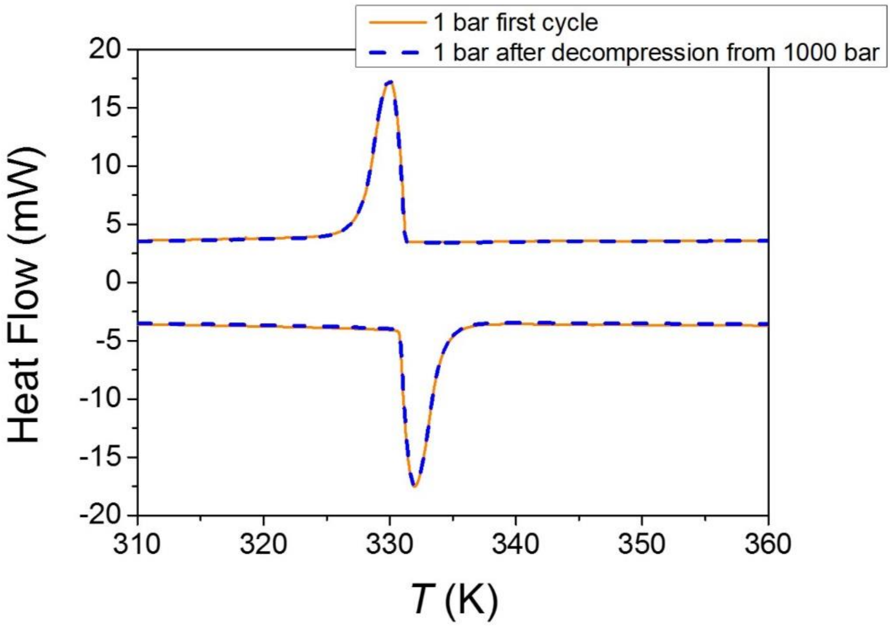

Supplementary Figure 3. Pressure cyclability of differential scanning calorimetry. Heat Flow (mW) versus temperature, T (K), at room pressure before (orange solid line) and after (blue dash line) applying hydrostatic pressure of 1000 bar.

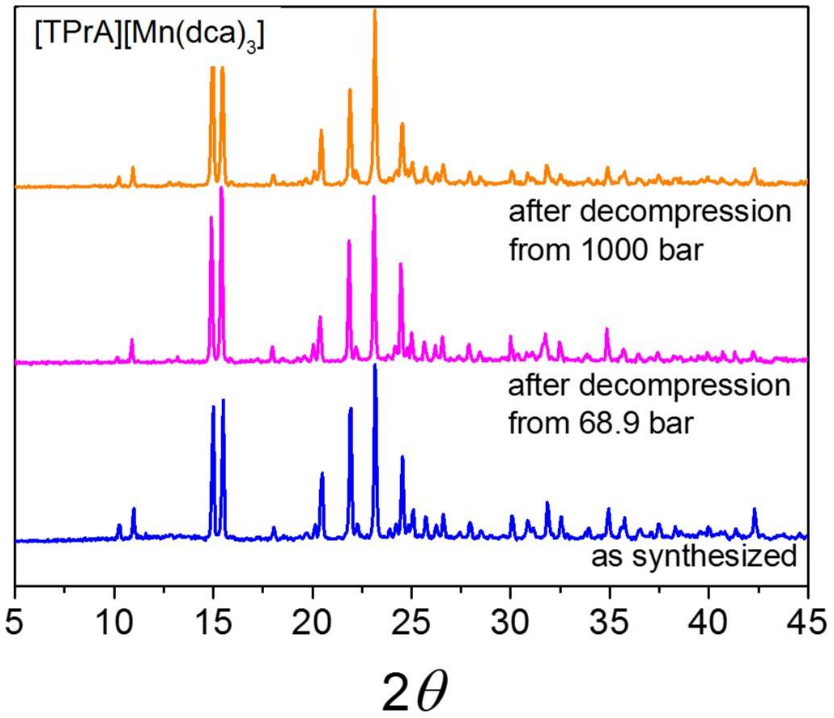

Supplementary Figure 4. **Powder X-ray diffraction.** PXRD patterns of the [TPrA][Mn(dca)$_3$] material as synthesized (bottom blue line) and after decompression from 68.9 bar (middle pink line) and 1000 bar (top orange line) in the PDSC analysis.

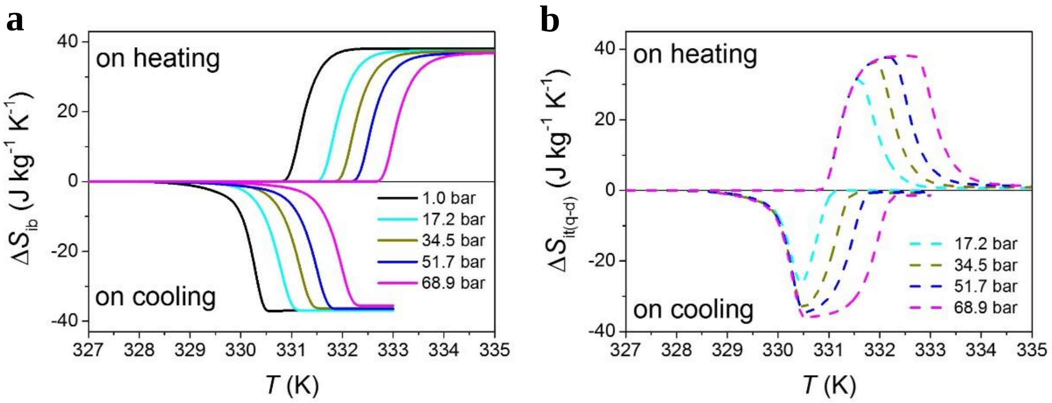

Supplementary Figure 5. Isobaric entropy change and barocalotic effect in the low pressure region. (a) Isobaric entropy change (ΔS_{ib}) as a function of temperature on heating (top curves) and cooling (bottom curves) and (b) barocaloric effect calculated as isothermal entropy change ($\Delta S_{\mathrm{it(q\text{-}d)}}$) on heating (top curves) and cooling (bottom curves) in the low pressure range (1 - 68.9 bar).

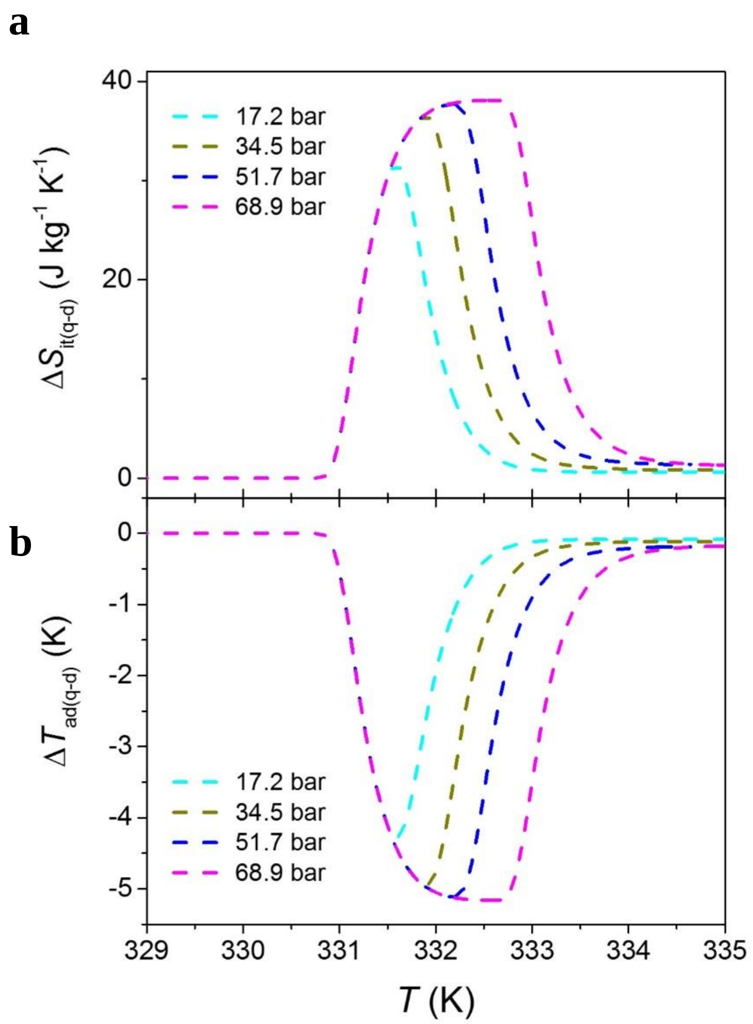

Supplementary Figure 6. Giant barocaloric effect observed on the heating curve. (a) Isothermal entropy change, $\Delta S_{\mathrm{it(q\text{-}d)}}$, and (b) adiabatic temperature change, $\Delta T_{\mathrm{ad(q\text{-}d)}}$, as a function of temperature at different applied hydrostatic pressures (from 1 bar to 68.9 bar).

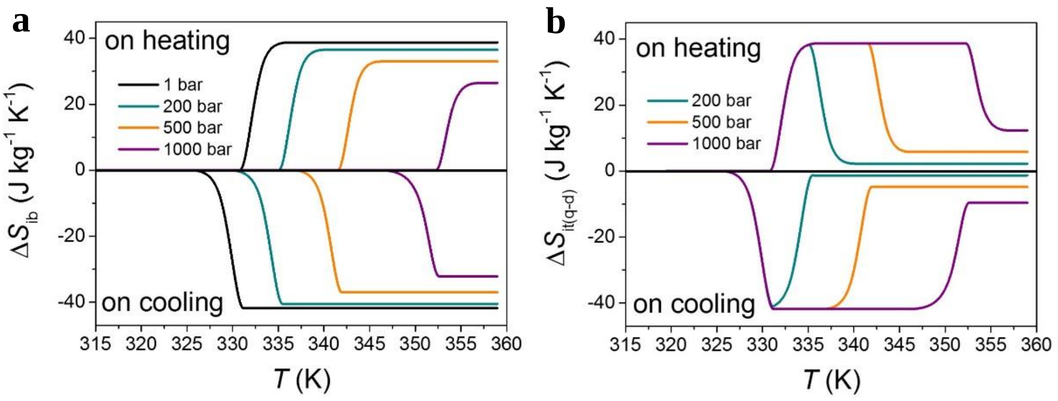

Supplementary Figure 7. Isobaric entropy change and barocalotic effect in the higher pressure region. (a) Isobaric entropy change (ΔS_{ib}) as a function of temperature on heating (top curves) and cooling (bottom curves) and (b) barocaloric effect calculated as isothermal entropy change ($\Delta S_{it(q\text{-}d)}$) on heating (top curves) and cooling (bottom curves) in the higher pressure range (1 - 1000 bar).

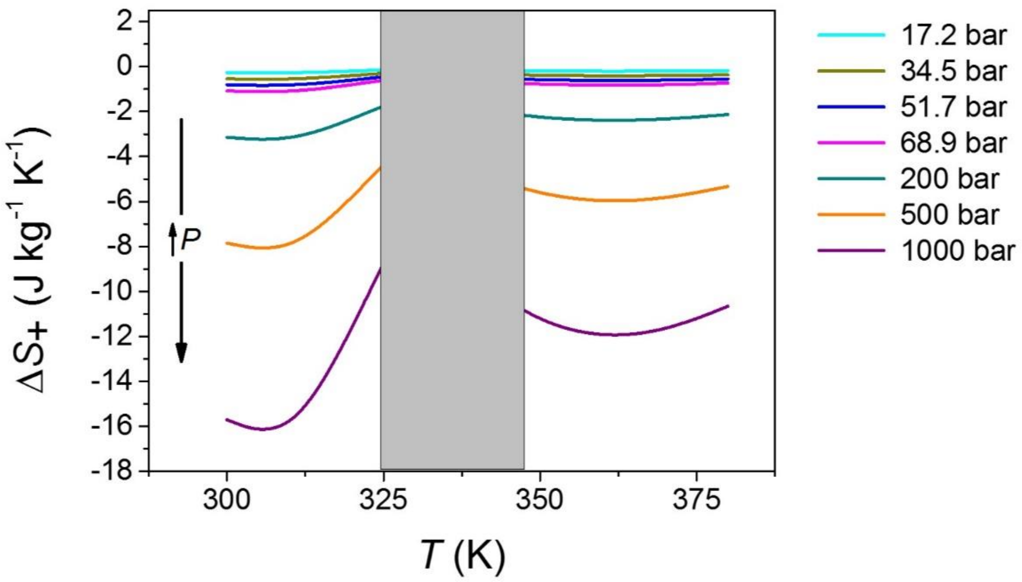

Supplementary Figure 8. Additional entropy change arising reversibly away from the first-order transition. Temperature dependence of $\Delta S_{+}(P) = -[m^{-1}(\delta V/\delta T)_{P=0}]P$ on applying pressure P, excluding the temperatures (grey zone) in which the pressure-dependent phase transition occurs.
